# Modulation of the PI3K/Akt Pathway and Bcl-2 Family Proteins Involved in Chicken’s Tubular Apoptosis Induced by Nickel Chloride (NiCl_2_)

**DOI:** 10.3390/ijms160922989

**Published:** 2015-09-23

**Authors:** Hongrui Guo, Hengmin Cui, Xi Peng, Jing Fang, Zhicai Zuo, Junliang Deng, Xun Wang, Bangyuan Wu, Kejie Chen, Jie Deng

**Affiliations:** 1 Key Laboratory of Animal Diseases and Environmental Hazards of Sichuan Province, Sichuan Agricultural University, Ya’an 625014, China; E-Mails: guohonrui@163.com (H.G.); pengxi197313@163.com (X.P.); fangjing4109@163.com (J.F.); zzcjl@126.com (Z.Z.); dengjl213@126.com (J.D.); wangxun99@163.com (X.W.); wubangyuan2008@163.com (B.W.); ckj930@126.com (K.C.); dengjl213@126.com (J.D.); 2 College of Veterinary Medicine, Sichuan Agricultural University, Ya’an 625014, China

**Keywords:** NiCl_2_, apoptosis, PI3K/Akt pathway, Bcl-2, mitochondria

## Abstract

Exposure of people and animals to environments highly polluted with nickel (Ni) can cause pathologic effects. Ni compounds can induce apoptosis, but the mechanism and the pathway of Ni compounds-induced apoptosis are unclear. We evaluated the alterations of apoptosis, mitochondrial membrane potential (MMP), phosphoinositide-3-kinase (PI3K)/serine-threonine kinase (Akt) pathway, and Bcl-2 family proteins induced by nickel chloride (NiCl_2_) in the kidneys of broiler chickens, using flow cytometry, terminal deoxynucleotidyl transferase 2ʹ-deoxyuridine 5ʹ-triphosphate dUTP nick end-labeling (TUNEL), immunohistochemstry and quantitative real-time polymerase chain reaction (qRT-PCR). We found that dietary NiCl_2_ in excess of 300 mg/kg resulted in a significant increase in apoptosis, which was associated with decrease in MMP, and increase in apoptosis inducing factor (AIF) and endonuclease G (EndoG) protein and mRNA expression. Concurrently, NiCl_2_ inhibited the PI3K/Akt pathway, which was characterized by decreasing PI3K, Akt1 and Akt2 mRNA expression levels. NiCl_2_ also reduced the protein and mRNA expression of anti-apoptotic Bcl-2 and Bcl-xL and increased the protein and mRNA expression of pro-apoptotic Bax and Bak. These results show that NiCl_2_ causes mitochondrial-mediated apoptosis by disruption of MMP and increased expression of AIF and EndoG mRNA and protein, and that the underlying mechanism of MMP loss involves the Bcl-2 family proteins modulation and PI3K/Akt pathway inhibition.

## 1. Introduction

Nickel (Ni) is one of the essential elements found in abundance in the earth’s crust occurring at an average concentration of about 75 μg/g [1]. Ni and Ni compounds have many industrial and commercial uses, and the progress of industrialization has led to their increased release into ecosystems [2,3]. Ni is considered an essential element in animals, microorganisms and plants, and is a constituent of enzyme proteins and nucleic acid [1,4]. However, symptoms of toxicity can occur when too much Ni is taken up [3]. Ni is potentially hazardous to living organisms due to its genotoxicity, immunotoxicity, mutagenicity and cancinogenicity [5,6,7]. Exposure of workers to Ni compounds can produce adverse effects on their health, such as Ni allergy, contact dermatitis, lung fibrosis, cardiovascular diseases, kidney diseases, and cancer of the respiratory tract [8]. Many forms of Ni may induce carcinoma in human beings and animals [9,10,11]. The findings of Zheng *et al.* [12] show that oxidative stress and the mitochondrial pathway play important roles in nickel sulfate (NiSO_4_)-induced apoptosis in *Carassius auratus* liver. Ni compounds can promote the generation of reactive oxygen species (ROS), interact directly or indirectly with nucleic acids and cause DNA damage [13]. It has been also suggested that nickel chloride (NiCl_2_) can induce DNA damage indirectly through the formation of ROS [14,15,16]. Efremenko *et al.* [17] have reported that nickel sulfide (Ni_3_S_2_)-caused inflammation and proliferation in the lungs of rats. Our previous studies have shown that dietary NiCl_2_ in excess of 300 mg/kg can cause immunotoxicity, oxidative damage and apoptosis in the kidneys, spleens, small intestines, and cecal tonsils of broiler chickens [18,19,20,21,22,23,24,25,26,27,28].

The phosphoinositide-3-kinase (PI3K)/serine-threonine kinase (Akt) signaling pathway plays a crucial role in cell growth and cell survival, and the pathway can be activated by many types of cellular stimuli or toxins [29]. Serine/threonine kinase Akt/PKB is the primary mediator of PI3K-initiated signaling. Akt, activated by PI3K, regulates cell survival through phosphorylation of a variety of downstream targets such as pro-apoptotic protein, transcription factors and another protein kinase [30,31]. The PI3K/Akt pathway can mediate cell-survival signals through the Bcl-2 family [4,32,33,34]. Among the Bcl-2 family proteins, Bcl-2 and Bcl-xL promote cell survival, while Bad, Bak, Bid, and Bax can induce cell death [35,36]. The Bcl-2 family of proteins, which is located on the mitochondrial membrane, can alter mitochondrial membrane permeability and trigger apoptosis [35,37,38], and Ni-induced apoptosis is reportedly associated with the PI3K/Akt pathway [4,33,34]. Wang *et al.* [39] suggest that nickel acetate induces cytotoxicity and apoptosis in HK-2 cells via ROS generation and that the mitochondria-mediated apoptotic signaling pathway is involved in the positive regulation of nickel acetate-induced renal cytotoxicity.

Although studies on apoptosis induced by Ni and Ni compounds have been reported, the mechanisms of Ni and Ni compounds-induced apoptosis are unclear. Therefore, the objective of this study was to determine potential mechanisms of NiCl_2_-induced mitochondria-mediated apoptosis in kidneys of broiler chickens and the alteration of the PI3K/Akt pathway and Bcl-2 family proteins. We monitored apoptosis, the change of mitochondrial membrane potential (MMP), and the mRNA expression of apoptosis inducing factor (AIF) and of endonuclease G (EndoG). We also measured the PI3K/Akt pathway (mRNA expression levels of PI3K, Akt1, Akt2) and the Bcl-2 family of proteins (the protein and mRNA expression of anti-apoptotic Bcl-2 and Bcl-xL and pro-apoptotic Bax and Bak).

## 2. Results

### 2.1. Histopathological Changes in the Kidney

**Figure 1:**
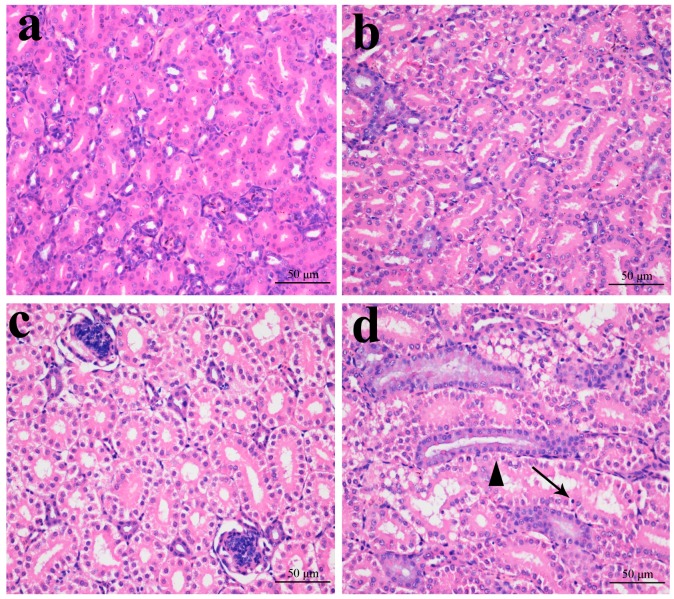
Histopathological changes in the kidney at 14 days of age. (**a**) Control group. No changes are observed (H·E × 400); (**b**) 300 mg/kg group. Tubular cells show slight granular degeneration (H·E × 400); (**c**) 600 mg/kg group. Tubular cells show granular degeneration and vacuolar degeneration (H·E × 400); and (**d**) 900 mg/kg group. Tubular cells show obvious granular and vacuolar degeneration. Also, few necrotic tubular cells (▲) and apoptotic tubular cells (↑) are observed (H·E × 400).

**Figure 2:**
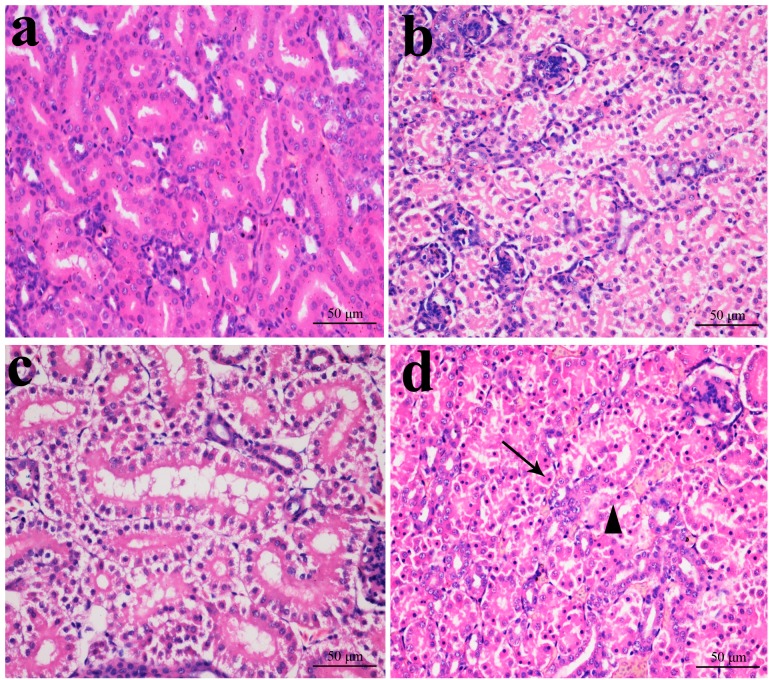
Histopathological changes in the kidney at 28 days of age. (**a**) Control group. No changes are observed (H·E × 400); (**b**) 300 mg/kg group. Tubular cells show granular degeneration (H·E × 400); (**c**) 600 mg/kg group. Tubular cells show obvious granular and vacuolar degeneration. Also, few necrotic tubular cells and apoptotic tubular cells are observed (H·E × 400); and (**d**) 900 mg/kg group. Tubular cells show marked granular and vacuolar degeneration. Also, some necrotic tubular cells (▲) and apoptotic tubular cells (↑) are observed (H·E × 400).

**Figure 3:**
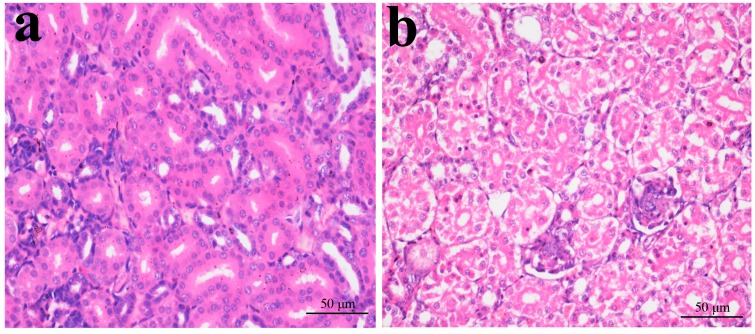

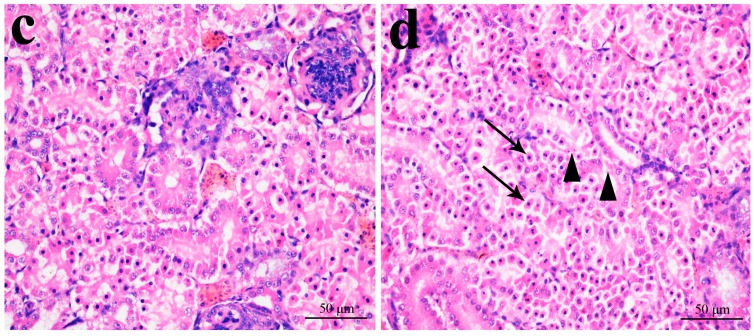
Histopathological changes in kidney at 28 days of age. (**a**) Control group. No changes are observed (H·E × 400); (**b**) 300 mg/kg group. Tubular cells show granular and vacuolar degeneration (H·E × 400); (**c**) 600 mg/kg group. Tubular cells show marked granular and vacuolar degeneration. Also, some necrotic tubular cells and apoptotic tubular cells are observed (H·E × 400); and (**d**) 900 mg/kg group. A large number of necrotic tubular cells (▲) and apoptotic tubular cells (↑) are observed (H·E × 400).

**Figure 4:**
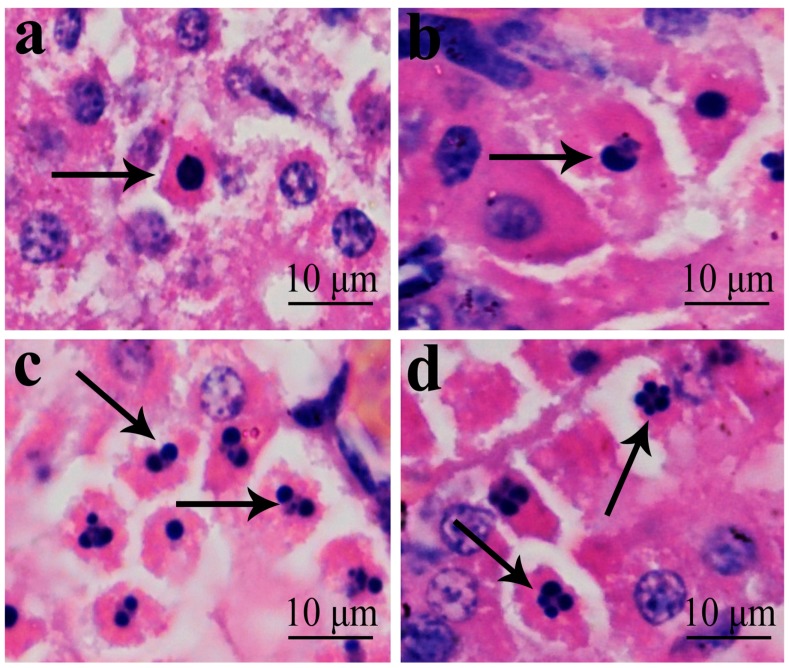
Morphological changes of apoptotic cells. (**a**) In the apoptotic cell, cytoplasm was intensely eosinophilic, and nucleus is shrunken and dense ring-shaped (↑). (H·E × 1000); and (**b**) In the apoptotic cell, nucleus is crescentic (↑). (H·E × 1000); (**c**) and (**d**) In the apoptotic cells, nuclei are cracked into two or multiple apoptotic bodies (↑). (H·E × 1000).

In Figure 1, Figure 2, Figure 3 and Figure 4, NiCl_2_ resulted in dose- and time-dependent histopathological changes in the kidney, including tubular granular degeneration, vacuolar degeneration, necrosis and apoptosis. In the granular and vacuolar degenerated tubular cells, tiny particles and small or large vacuoles appeared in the cytoplasm. Karyorrhexis, karyolysis and hypochromatosis appeared in the necrotic cells. In the apoptotic cells, cytoplasm was intensely eosinophilic, and nuclei were shrunken, dense, ring-shaped and crescentic. Apoptotic bodies were also observed.

### 2.2. Effects of NiCl_2_ on Apoptosis in the Kidney

**Figure 5:**
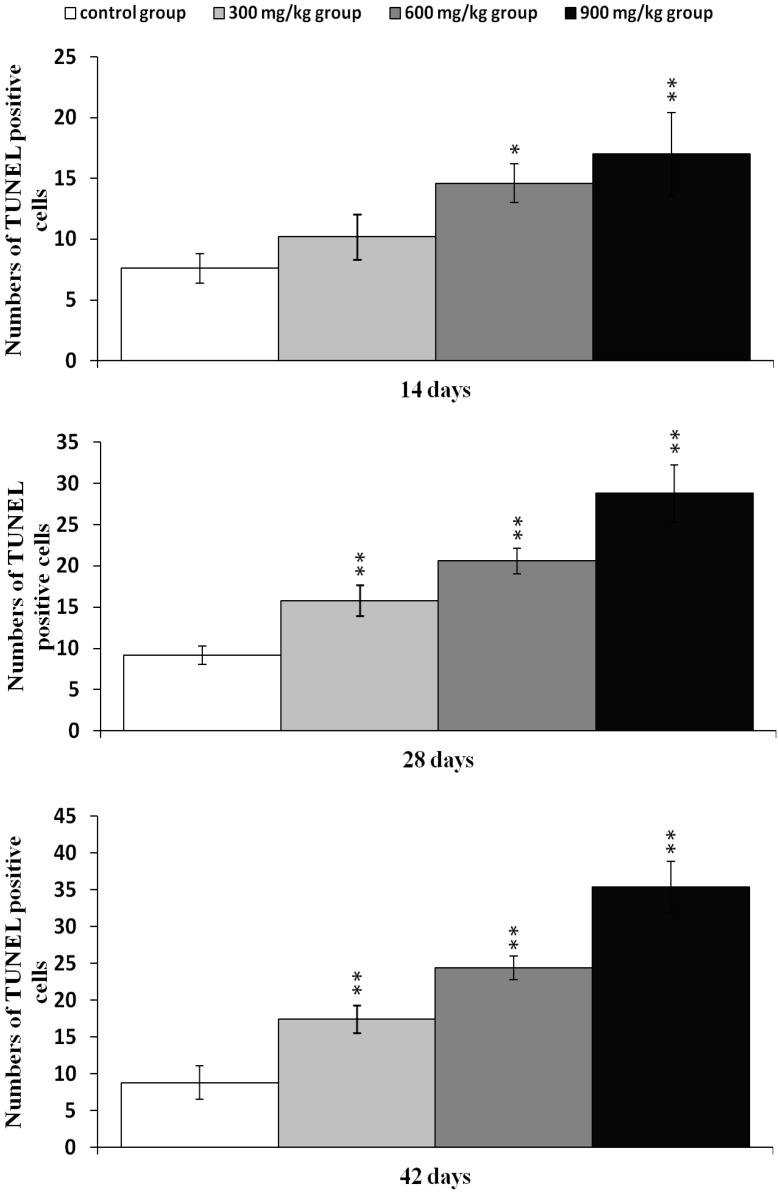
Changes of TUNEL-positive cells at 14, 28 and 42 days. Data are presented with the mean ± standard deviation (*n* = 5 × 5); * *p* < 0.05, compared with the control group; ** *p* < 0.01, compared with the control group.

The effects of dietary NiCl_2_ on the apoptosis in the kidney were studied with TUNEL assay. The results presented in Figure 5, showed that the number of apoptotic cells was significantly greater (*p* < 0.05 or *p* < 0.01) in the 600 and 900 mg/kg groups at 14 days of age than in the control group. Apoptotic cells were significantly increased (*p* < 0.05 or *p* < 0.01) also in the three NiCl_2_-treated groups at 28 to 42 days of age.

### 2.3. Effects of NiCl_2_ on MMP, and AIF and EndoG Protein and mRNA Expression in the Kidney

To further confirm the role of mitochondria in NiCl_2_-induced apoptosis, the changes of the MMP and release of AIF and EndoG protein from the mitochondria to the nucleus were examined.

**Figure 6:**
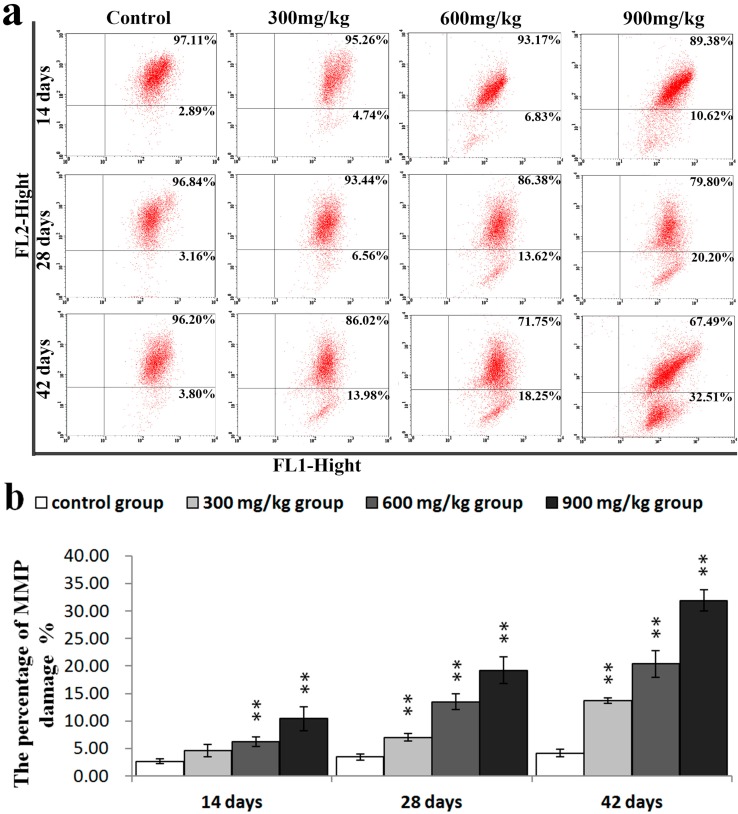
NiCl_2_-induced mitochondrial dysfunction in the kidney. (**a**) Representative flow cytometric diagram of MMP analysis; and (**b**) The percentage of MMP damage. Data are presented with the mean ± standard deviation (*n* = 5); ** *p* < 0.01, compared with the control group.

As illustrated in Figure 6a,b, the results of the flow cytometry assay showed that NiCl_2_ caused a significant loss (*p* < 0.05 or *p* < 0.01) in the MMP of the three NiCl_2_-treated groups from 28 to 42 days of age and in the 600 and 900 mg/kg groups at 14 days of age as, compared with that in the control group.

**Figure 7:**
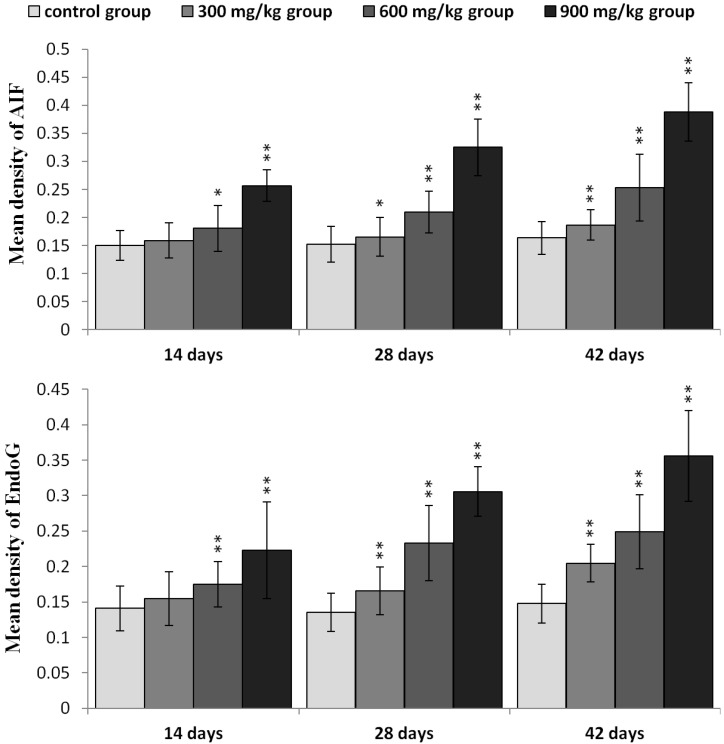
The protein expression levels of AIF and EndoG in the kidney. Data are presented with the mean ± standard deviation (*n* = 5 × 5); * *p* < 0.05, compared with the control group; ** *p* < 0.01, compared with the control group.

In Figure 7, AIF and EndoG protein expression was significantly higher (*p* < 0.05 or *p* < 0.01) in the three NiCl_2_-treated groups from 28 to 42 days of age and in the 600 and 900 mg/kg group at 14 days of age, than in the control group.

**Figure 8:**
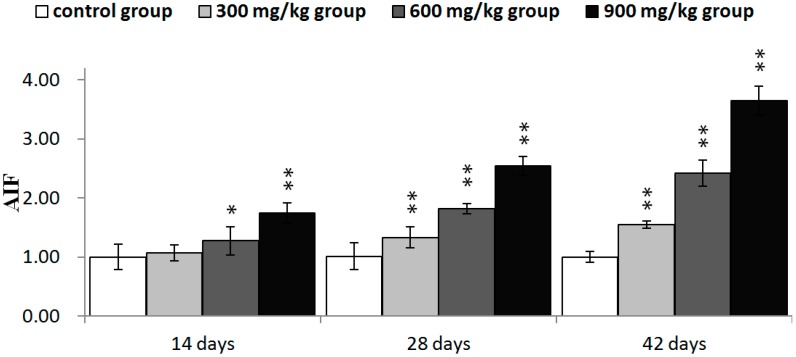

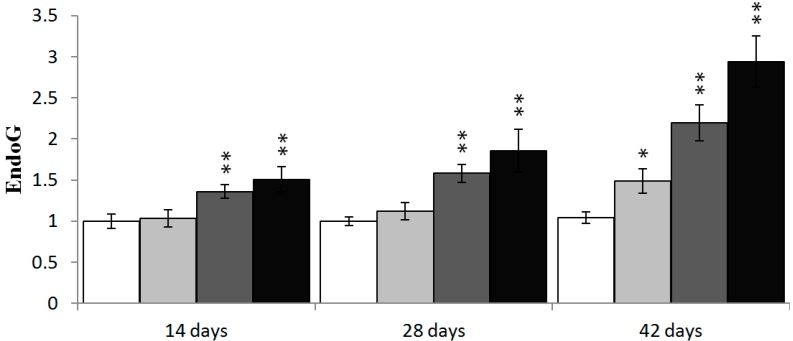
The mRNA expression levels of AIF and EndoG in the kidney. Data are presented with the mean ± standard deviation (*n* = 5); * *p* < 0.05, compared with the control group; ** *p* < 0.01, compared with the control group.

AIF mRNA expression was significantly higher (*p* < 0.05 or *p* < 0.01) in the three NiCl_2_-treated groups from 28 to 42 days of age and in the 600 and 900 mg/kg group at 14 days of age, than in the control group. EndoG mRNA expression was significantly higher (*p* < 0.05 or *p* < 0.01) in the 600 and 900 mg/kg group from 14 to 42 days of age and in the 300 mg/kg group at 42 days of age than in the control group, as shown in Figure 8.

### 2.4. Effects of NiCl_2_ on Phosphoinositide-3-Kinase (PI3K)/Serine-Threonine Kinase (Akt) Pathway in the Kidney

**Figure 9:**
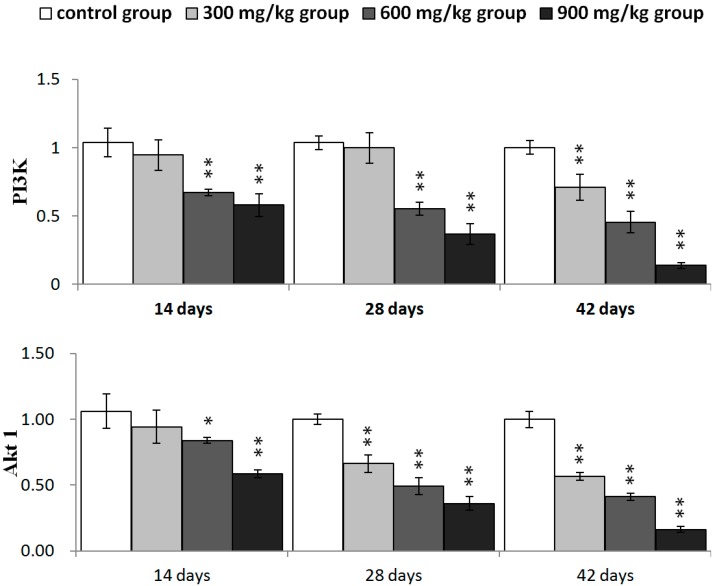

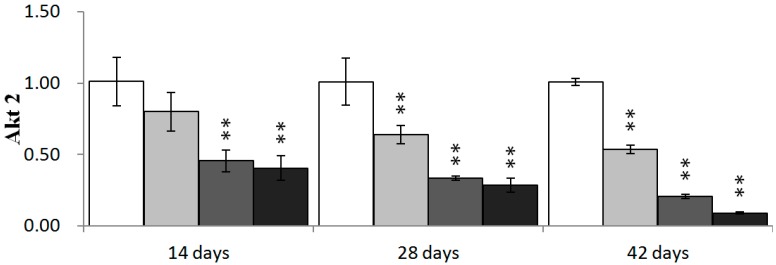
The mRNA expression levels of PI3K, Akt1 and Akt2 in the kidney. Data are presented with the mean ± standard deviation (*n* = 5); * *p* < 0.05, compared with the control group; ** *p* < 0.01, compared with the control group.

We investigated whether PI3K/Akt was involved in NiCl_2_-mediated apoptosis. As shown in Figure 9, PI3K mRNA expression was significantly decreased (*p* < 0.05 or *p* < 0.01) in the 600 and 900 mg/kg groups from 14 to 42 days of age and in the 300 mg/kg group at 42 days of age. The mRNA expression of Akt1 and Akt2 was significantly lower (*p* < 0.05 or *p* < 0.01) in the three NiCl_2_-treated groups from 28 to 42 days of age and in the 600 and 900 mg/kg groups at 14 days of age than in the control group.

### 2.5. Expression of NiCl_2_ on Bcl-2 Family Protein and mRNA Expression in the Kidney

It has been suggested that Bcl-2 can be a crucial mediator downstream of PI3K/Akt signaling. And Bcl-2 famly proteins have been shown to regulate the MMP. Therefore, we examined the effect of NiCl_2_ treatment on Bcl-2 family proteins in the kidney.

**Figure 10:**
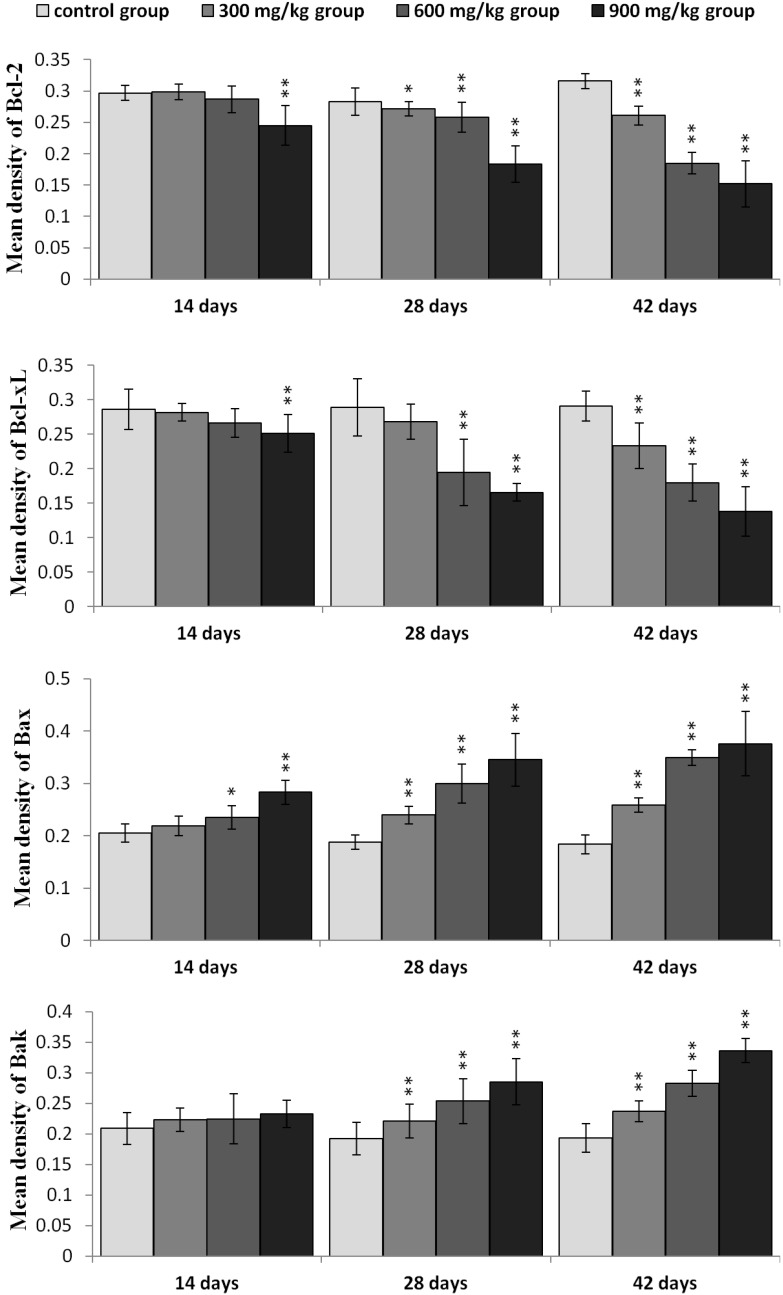
The protein expression levels of Bcl-2, Bcl-xL, Bax and Bak in the kidney. Data are presented with the mean ± standard deviation (*n* = 5 × 5); * *p* < 0.05, compared with the control group; ** *p* < 0.01, compared with the control group.

In Figure 10, Bcl-2 protein expression was significantly decreased (*p* < 0.05 or *p* < 0.01) in the three NiCl_2_-treated groups from 28 to 42 days of age and in the 900 mg/kg group at 14 days of age when compared with those in the control group. Bcl-xL protein expression was significantly decreased (*p* < 0.05 or *p* < 0.01) in the 900 mg/kg group at 14 days of age, in the 600 and 900 mg/kg groups at 48 days of age and in the three NiCl_2_-treated groups at 42 days of age. The protein expression of Bax and Bak was significantly increased (*p* < 0.05 or *p* < 0.01) in the three NiCl_2_-treated groups from 28 to 42 days of age. And, Bax protein expression was significantly increased (*p* < 0.05 or *p* < 0.01) in the 600 and 900 mg/kg groups at 14 days of age when compared with those in the control group.

**Figure 11:**
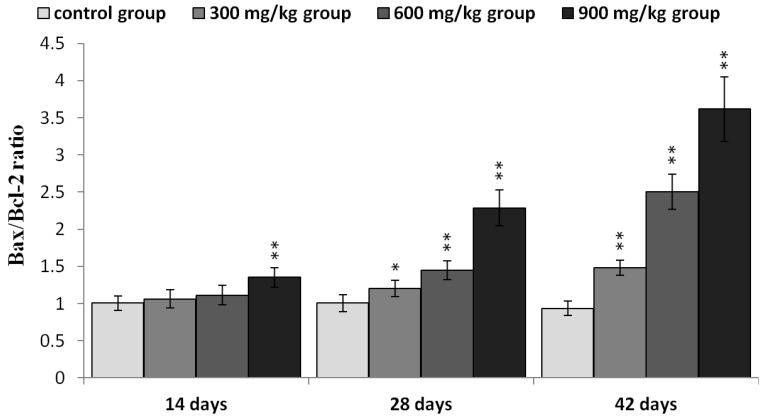
The ratio of Bax/Bcl-2 protein expression in the kidney. Data are presented with the mean ± standard deviation (*n* =5 × 5); * *p* < 0.05, compared with the control group; ** *p* < 0.01, compared with the control group.

The protein expression of Bax/Bcl-2 ratio was significantly higher (*p* < 0.05 or *p* < 0.01) in the three NiCl_2_-treated groups from 28 to 42 days of age and in the 900 mg/kg groups from 14 days of age than in the control group, as shown in Figure 11.

**Figure 12:**
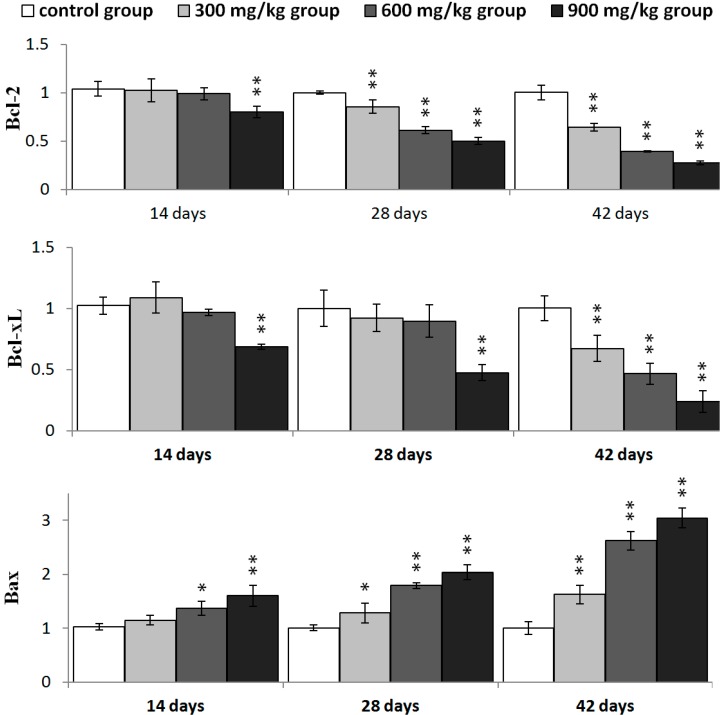

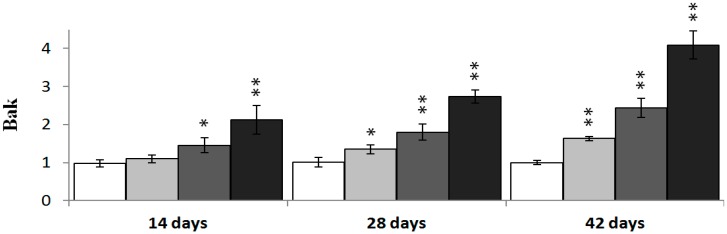
The mRNA expression levels of Bcl-2, Bcl-xL, Bax and Bak in the kidney. Data are presented with the mean ± standard deviation (*n* = 5); * *p* < 0.05, compared with the control group; ** *p* < 0.01, compared with the control group.

In Figure 12, Bcl-2 mRNA expression was significantly decreased (*p* < 0.05 or *p* < 0.01) in the three NiCl_2_-treated groups from 28 to 42 days of age and in the 900 mg/kg group at 14 days of age. Bcl-xL mRNA expression was significantly lower (*p* < 0.05 or *p* < 0.01) in the three NiCl_2_-treated groups from 42 days of age and in the 900 mg/kg group from 14 to 28 days of age than that in the control group. The mRNA expression of Bax and Bak was significantly increased (*p* < 0.05 or *p* < 0.01) in the three NiCl_2_-treated groups from 28 to 42 days of age and in the 600 and 900 mg/kg groups at 14 days of age.

**Figure 13:**
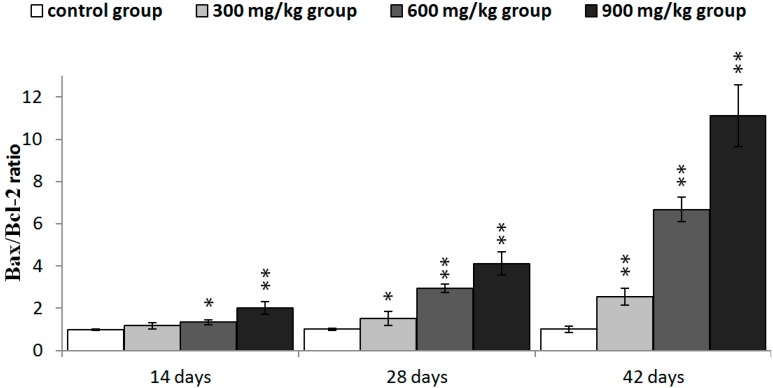
The ratio of Bax/Bcl-2 mRNA expression in the kidney. Data are presented with the mean ± standard deviation (*n* = 5); * *p* < 0.05, compared with the control group; ** *p* < 0.01, compared with the control group.

The Bax/Bcl-2 ratio was significantly higher (*p* < 0.05 or *p* < 0.01) in the three NiCl_2_-treated groups from 28 to 42 days of age and in the 600 and 900 mg/kg groups from 14 days of age than in the control group, as shown in Figure 13.

## 3. Discussion

This study explores the molecular control pathways of dietary NiCl_2_-induced apoptosis in the kidney of young chickens. We found consistent evidence that dietary NiCl_2_ in excess of 300 mg/kg had adverse effects on the kidney cells. The results showed that NiCl_2_ significantly increased the TUNEL-postive cells, which were regarded as the apoptotic cells. The histopathological changes also showed that NiCl_2_ increased apoptosis in the kidney. Our previous studies have proved that NiCl_2_ induced apoptosis in the thymus, cecal tonsil and spleen [18,21,40]. Also, our findings are in agreement with the the results of Ma *et al.* and Hossain *et al.* [41,42] who demonstrate that nickel nanowires (Ni NWs) induce apoptosis in Hela cells and human pancreatic adenocarcinoma cells. Also, Gathwan *et al.* [43] have reported that NiCl_2_ can cause apoptotis in liver of male mice.

Mitochondria play an important role in the regulation of cell apoptosis [35]. Changes in the MMP are considered an early event in apoptosis and many proapoptotic proteins can be released from the mitochondria into the cytoplasm when the MMP is damaged [44]. We investigated whether mitochondria were involved in NiCl_2_-induced apoptosis in broilers. As shown by flow cytometry analysis, NiCl_2_ reduced the MMP in the kidney. Moreover, AIF and EndoG protein and mRNA expression were increased after disruption of the MMP. AIF and EndoG are thought to be mitochondrial cysteine proteases, whose release can be blocked by Bcl-2. After the MMP is damaged, AIF and EndoG can translocate from mitochondria to the nucleus, and there cause DNA fragmentation and cleavage of genomic DNA without being activated by caspases [45,46]. The data here shows that NiCl_2_ induces apoptosis through a mitochondria-mediated pathway in the kidney. Previous studies show that nickel acetate can leads to apoptosis via a mitochondrial-mediated pathway [39]. Also, Zhao *et al.* [47] have reported that nickel nanoparticles (Ni NPs) and nickel fine particles induce changes in the MMP, and up-regulate and mobilize AIF from mitochondria to cytoplasm in JB6 cells.

Recently, it has been reported that regulation of mitochondrial-mediated apoptosis requires the involvement of the Bcl-2 family proteins [48]. The anti-apoptotic members of the Bcl-2 family proteins tend to stabilize the barrier function of mitochondrial membranes, whereas pro-apoptotic members of Bcl-2 family proteins destabilize this function [36]. Loss of MMP is a prerequisite for mitochondrial-mediated apoptosis as it is associated with the reshuffling of Bcl-2 family proteins [49]. The ratio between anti- and pro-apoptotic proteins is a determinant of tissue homeostasis because it influences the sensitivity of cells to apoptosis [35,50]. In this study, NiCl_2_ increased pro-apoptotic Bax and Bak protein and mRNA expression, and concomitantly decreased anti-apoptotic Bcl-2, Bcl-xL protein and mRNA expression, which caused a significant increase in the Bax/Bcl-2 ratio, and then promoted apoptosis. Our previous studies have also shown that NiCl_2_ increases the mRNA expression levels of Bax and decreases the mRNA expression levels of Bcl-2 in the thymus, cecal tonsil and spleen [18,21,40].

The PI3K/Akt pathway also plays a role in the regulation of Bcl-2 family proteins, which are believed to be important targets for anti-apoptosis [29,51]. The activation of PI3K/Akt pathway leads to increase in Bcl-2, Bcl-xL expression, and decrease in Bad, Bax expression [30,52,53,54]. We have investigated whether the PI3K/Akt pathway is involved in NiCl_2_-induced mitochondria-mediated apoptosis. The results showed that dietary NiCl_2_ reduced the expression of PI3K and Akt mRNA, implying that inhibition of the PI3K/Akt pathway is one mechanism of NiCl_2_-induced apoptosis. This implication is consistent with the report of Liu *et al.* [55] that NiCl_2_ induces apoptosis and decreases PI3K, p-Akt and Bcl-2 protein expression and increases Bax protein expression in the liver of Kunming mice. Our results are also in agreement with the results of Wang *et al.* [39] in which nickel acetate-induced apoptosis is characterized by decreasing the protein expression of Bcl-2 and Bcl-xL and increasing the protein expression of Bad, Bcl-Xs, Bax, cytochrome c and caspases 9, 3 and 6 in human proximal tubule cells. Pan *et al.* [56] suggest that Ni_3_S_2_ can down-regulate several anti-apoptotic proteins, including Bcl-2 and Bcl-xL in human bronchial epithelial (BEAS-2B) cells. Contrary to our results and the above-mentioned references, Shi [54] suggests that NiCl_2_ up-regulates expression of Akt, Bcl-2, and Bcl-_X_L in BEAS-2B cells.

**Figure 14:**
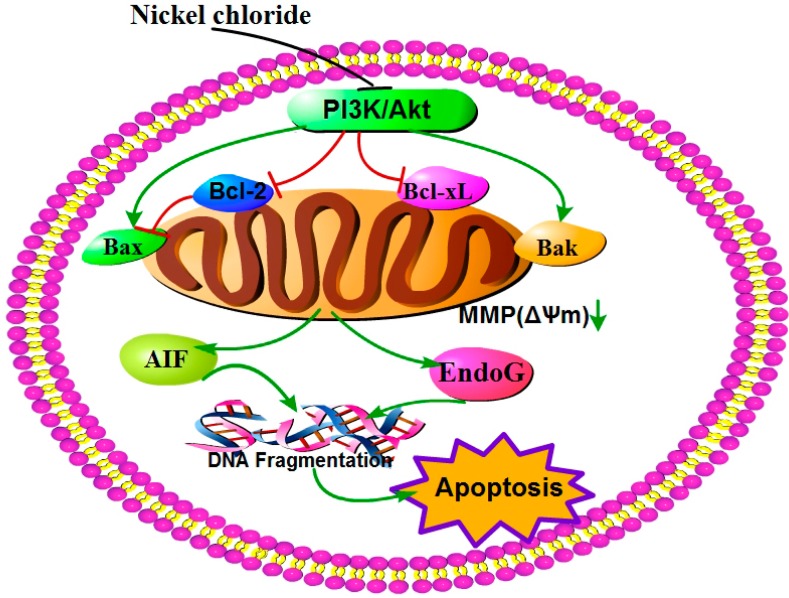
Schematic diagram of NiCl_2_-caused mitochondria-mediated apoptosis.

Based on the results of our study and the discussion above, the mechanism of NiCl_2_-caused mitochondria-mediated apoptosis in the kidney is summarized in Figure 14.

NiCl_2_ modulates the Bcl-2 family proteins and inhibits the PI3K/Akt pathway. This action is followed by MMP disruption, which increases AIF and EndoG protein and mRNA expression. The highly expressed AIF and EndoG translocate from mitochondria to the nucleus and there causes DNA damage, which finally leads to renal-cell apoptosis.

## 4. Experimental Section

### 4.1. Animals and Treatment

Two hundred and eighty one-day-old healthy broilers (Chia Tai Group, Wenjiang, Sichuan, China) were divided into four groups. There were seventy broilers in each group. Broilers were housed in cages with electrical heaters, and provided with water as well as under-mentioned experimental diets *ad libitum* for 42 days. The growth cycle of commercial broilers is about 42 days, after which they are used for consumption. In this rapid growth period food consumption is high, and broilers will easily be affected by diet containing metal pollutants (such as Ni). The aim of our study is to evaluate the effect of dietary NiCl_2_ on the broilers in this period of rapid growth.

To observe the time-dependent dynamic change, we chose three time points (14, 28 and 42 days of age) for examining histopathological injury, the alterations of apoptosis, mitochondrial membrane potential (MMP), apoptotic protein expression and mRNA expression levels.

In this study, a corn-soybean basal diet formulated by the National Research Council [57] was the control diet. NiCl_2_ (NiCl_2_·6H_2_O, ChengDu Kelong Chemical Co., Ltd., Chengdu, China) was mixed into the corn-soybean basal diet to produce the experimental diets containing 300, 600 and 900 mg/kg NiCl_2_, respectively.

The basis of doses (300, 600 and 900 mg/kg NiCl_2_) selection: Ling and Leach reported that dietary NiCl_2_ concentrations of 300 mg/kg and over resulted in significant reduction in growth rate. Mortality and anemia were observed in chicks receiving 1100 mg/kg nickel [58]. Weber and Reid found a significant growth reduction at 700 mg/kg NiSO_4_ and nickel acetate and over [59]. Chicks fed more than 250–300 mg/kg Ni in the diet exhibited depressed growth and reduced feed intake [60]. Bersenyi *et al.* [61] reported that supplementation of 500 mg/kg NiCl_2_ reduced weight gain (by 10%), feed intake (by 4%) and worse feed conversion efficiency (FCE) (by 5%) in growing broiler cockerels. According to the above-mentioned research results and our preliminary experiment, we chose the doses of 300, 600 and 900 mg/kg NiCl_2_ in this study for observing the does-dependent changes.

Our experiments involving the use of broilers, and all experimental procedures were approved by Animal Care and Use Committee, Sichuan Agricultural University (Approval No: 2012-024).

### 4.2. Histopathological Examination of Kidney

Five chickens in each group were humanely killed at 14, 28 and 42 days of age. Kidneys were removed, fixed in 4% paraformaldehyde, dehydrated in ethanol and embedded in paraffin. Serial slices at 5 mm thickness were prepared and stained with haematoxylin and eosin (H&E), and examined by light microscopy.

### 4.3. Detection of Renal Apoptosis by TUNEL

Five broilers in each group were humanely sacrificed at 14, 28, and 42 days of age. Kidneys were removed, fixed in 4% paraformaldehyde, dehydrated in ethanol and embedded in paraffin.

TUNEL analysis was carried out according to the manual of In Situ Cell Death Detection Kit (Cat: 11684817980, Roche, Mannheim, Germany). Briefly, tissue sections (5 μm thick) were rehydrated in a series of xylene and ethanol solutions and then rinsed in ddH_2_O, digested with 50 μL proteinase K (diluted in Tris·HCl pH 7.8) for 15 min, and incubated with 3% H_2_O_2_ in methanol for 15 min at room temperature to inactivate endogenous peroxidase. The sections were transferred to a reaction mixture containing biotin-dUTP terminal deoxynucleotidyl and incubated in a humidified chamber for 1 h at 37 °C, followed by washing in phosphate buffer saline (PBS), pH 7.2–7.4. Sections were incubated in Converter-POD (HRP) for 30 min at 37 °C. Reaction product was visualized with DAB kit (AR1022, Boster, Wuhan, China). After final washing in ddH_2_O, slices were lightly counterstained with hematoxylin, dehydrated in ethanol, cleared in xylene and mounted.

Cells were observed with light microscopy (Olympus, Shimadzu, Japan). The nuclei of apoptotic cells containing DNA strand breaks were stained brown. The TUNEL positive cells (apoptotic cells) were counted by use of a computer-supported imaging system connected to a light microscope with an objective magnification of 1000×. Apoptotic cells were quantified by use of Image-Pro Plus 5.1 (Madia Cybernetics, Bethesda, MD, USA) image analysis software. Five sections in each chicken and five fields in each section were measured and averaged.

### 4.4. Detection of Mitochondrial Membrane Potential (ΔѰm) in the Kidney by Flow Cytometry

Mitochondrial membrane potential was measured with a mitochondrial membrane potential detection kit (Cat: 551302, Lot: 3242965, BD, Franklin lakes, NJ, USA).

At 14, 28 and 42 days of age, five broilers in each group were humanely killed, and the kidneys were immediately removed and ground to form a cell suspension, which was filtered through a 300-mesh nylon screen. The cells were washed twice with ice-cold PBS (pH 7.2–7.4) and suspended in PBS at a concentration of 1 × 10^6^ cells/mL. One milliliter of the cell suspension was transferred to a 5-mL culture tube. The collected cells were incubated with JC-1 (5,5ʹ,6,6ʹ-tetra-chloro-1,1ʹ,3,3ʹ-tetra-ethylbenzimidalyl-carbocyanineiodide) working solution for 15 min in a 37 °C, 5% CO_2_ incubator. The staining solution was removed and the cells were washed twice with JC-1 staining buffer. The cell-associated fluorescence was measured with flow cytometry (BD, Franklin Lakes, NJ, USA). Normal ΔΨm produces red fluorescence for JC-1 aggregates, but loss of the ΔΨm results in the disaggregation of JC-1 and produces green fluorescence.

### 4.5. Detection of Protein Expression in the Kidney by Immunohistochemistry

Five chickens in each group were humanely sacrificed for gross examination at 14, 28 and 42 days of age. Kidneys were collected and fixed in 10% neutral buffered formalin, and then processed, trimmed, and embedded in paraffin wax.

The method used was that described by Wu *et al.* [21]. Tissue slices were dewaxed in xylene, rehydrated through a graded series of ethanol solutions, washed in distilled water and PBS and endogenous peroxidase activity was blocked by incubation with 3% H_2_O_2_ in methanol for 15 min. The sections were subjected to antigen retrieval procedure by microwaving in 0.01 M sodium citrate buffer pH 6.0. Additional washing in PBS was performed before 30 min of incubation at 37 °C in 10% normal goat serum (Boster). The slices were incubated overnight at 4 °C with anti-Bax (1:400) (Cat: 14796, Cell Signaling Technology, Danvers, MA, USA); anti-Bak (1:400) (Cat: 12105, Cell Signaling Technology); anti-Bcl-xL (1:300) (Cat: 2764, Cell Signaling Technology, anti-Bcl-2 (1:400) (Cat: 15071, Cell Signaling Technology); anti-AIF(1:200) (Cat: sc-9416, Santa Cruz Biotechnology, Dallas, TX, USA); anti-EndoG (1:100), (Cat: orb6003, Biorbyt, San Francisco, CA, USA). After washing in PBS, the slices were exposed to 1% biotinylated goat anti-mouse IgG secondary antibody (Boster) for 1 h at 37 °C, and then incubated with strept avidin-biotin complex (SABC; Boster) for 30 min at 37 °C. To visualize the immunoreaction, sections were immersed in diaminobenzidine hydrochloride (DAB; Boster). The slices were monitored microscopically and stopped by immersion in distilled water, as soon as brown staining was visible. Slices were lightly counterstained with hematoxylin, dehydrated in ethanol, cleared in xylene and mounted.

The protein expression levels were measured using a computer-supported imaging system connected to a light microscope (OlympusAX70) with an objective magnification of 400×. The intensity of staining for each protein was quantified using Image-pro Plus 5.1 (Madia Cybernetics, Bethesda, MD, USA). Each group was measured in five sections and each section was measured using five views, and averaged.

### 4.6. Detection of mRNA Expression in the Kidney by qRT-PCR

The kidneys from five chickens in each group were taken at 14, 28, and 42 days of age and stored in liquid nitrogen. They kidneys were homogenized in liquid nitrogen with a mortar and pestle.

As described [21], total RNA was extracted from the frozen kidney powders with RNAiso Plus (9108/9109, Takara, Dalian, China) according to the manufacturer’s protocol. Next, cDNA was synthesized with a Prim-Script™ RT reagent Kit (RR047A, Takara, Japan) according to the manufacturer’s protocol. The cDNA product was used as a template for qRT-PCR analysis. Sequences for target genes were obtained from the NCBI database. Oligonucleotide primers were designed by use of Primer 5 software and synthesized at Takara (Dalian, China), as shown in Table 1.

**Table 1:** A list of primers in qRT-PCR analysis of mRNA expression of the apoptotic proteins.

Gene Symbol	Accession Number	Primer	Primer Sequence (5ʹ–3ʹ)	Product Size (bp)	Tm (°C)
PI3K	NM001004410	F	CGGATGTTGCCTTACGGTTGT	162	58
		R	GTTCTTGTCCTTGAGCCACTGAT		
Akt1	AF039943	F	TGATGGCACATTCATTGGCTAC	122	58
		R	TGTTTGGTTTAGGTCGTTCTGTCT		
Akt2	AF181260	F	CCGAAGTGCTGGAGGACAAC	115	60
		R	CGCTCGTGGTCCTGGTTGTA		
Bcl-2	NM205339	F	GATGACCGAGTACCTGAACC	114	61
		R	CAGGAGAAATCGAACAAAGGC		
Bax	XM422067	F	TCCTCATCGCCATGCTCAT	69	62
		R	CCTTGGTCTGGAAGCAGAAGA		
Bak	NM001030920	F	TCTACCAGCAAGGCATCACGG	122	60
		R	ATCGAGTGCAGCCACCCATC		
Bcl-xL	GU230783	F	ATGAGTTTGAGCTGAGGTACCGG	150	59
		R	AGAAGAAAGCCACGATGCGC		
AIF	NM001007490	F	CTGGGTCCTGATGTGGGCTAT	123	58
		R	TGTCCCTGACTGCTCTGTTGC		
EndoG	XM415487	F	TGCCTGGAATAACCTTGAGAAATAC	170	61
		R	TGAAGAAATGGGTAGGGACGG		
β-actin	L08165	F	TGCTGTGTTCCCATCTATCG	178	62
		R	TTGGTGACAATACCGTGTTCA		

All qRT-PCR were performed by use of the SYBR^®^ Premix Ex Taq™ II system (DRR820A, Takara, Japan) with on a Model C1000 Thermal Cycler (Bio Rad, Hercules, CA, USA).

Chicken β-actin expression was used as an internal reference housekeeping gene. Gene expression values from control group subsamples at 14, 28, and 42 days of age were used to calibrate gene expression in subsamples from corresponding experimental subsamples. All data output from the qRT-PCR experiments were analyzed by use of the 2^−ΔΔ*C*t^ method [62].

### 4.7. Statistical Analysis

The significance of difference among the four groups of broiler chicks was assessed with variance analysis, and results were presented as mean ± standard deviation (M ± SD). The variation was measured by use of one-way analysis of variance (ANOVA) test of SPSS 16.0 for windows. *p* < 0.05 was considered statistical significance.

## 5. Conclusions

In chicks, dietary NiCl_2_ in excess of 300 mg/kg was found to cause mitochondrial-mediated apoptosis by disruption of MMP and increased expression of AIF and EndoG mRNA and protein. These results indicate that the underlying mechanism of MMP disruption involves Bcl-2 family protein modulation and PI3K/Akt pathway inhibition.
